# Supplementary material for: Splice-Junction-Based Mapping of Alternative Isoforms in the Human Proteome
Source: Cell Rep. Author manuscript; Available in PMC 2020 Jan 15. (PMC6961840; doi:10.1016/j.celrep.2019.11.026)
Supplement: 2 [file NIHMS1546469-supplement-2.zip › DF1/PXD009737/TestisTrypsin/TestisTrypsin_31_CADM1_AHSDYMLYVYDTTATTEPAVHDSR.pdf]

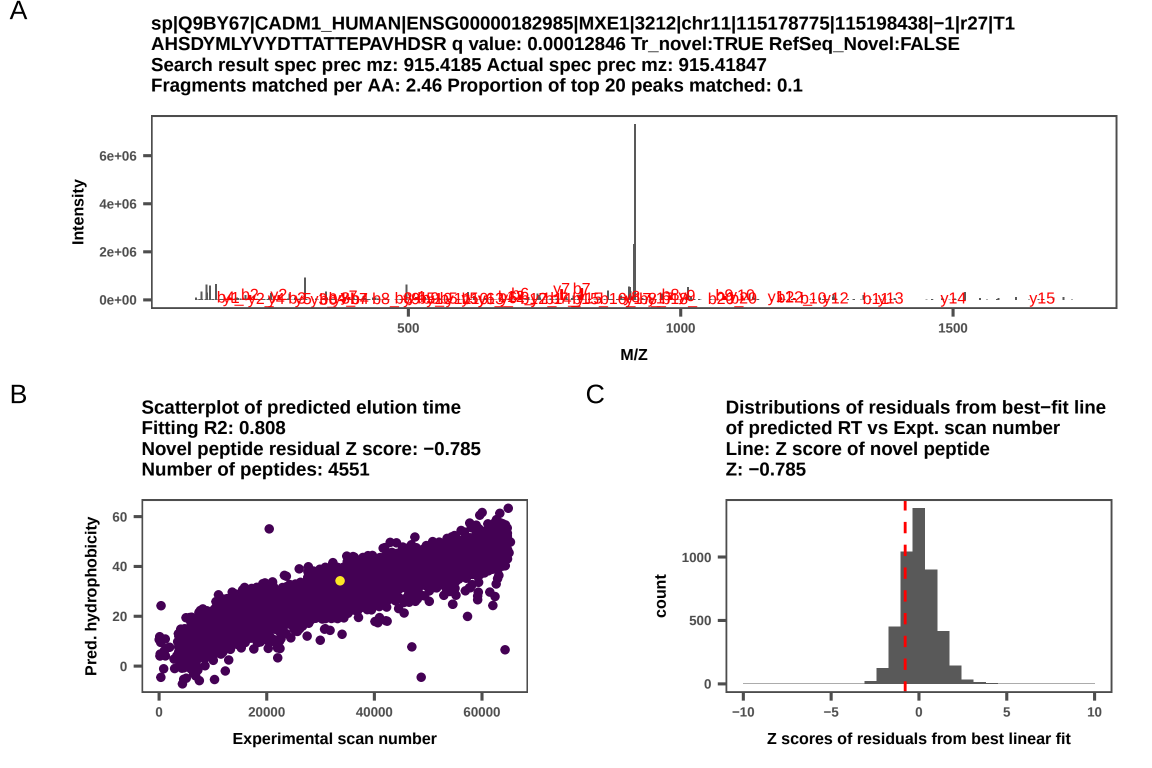
A
sp|Q9BY67|CADM1_HUMAN|ENSG00000182985|MXE1|3212|chr11|115178775|115198438|−1|r27|T1
AHSDYMLYVYDTTATTEPAVHDSR q value: 0.00012846 Tr_novel:TRUE RefSeq_Novel:FALSE
Search result spec prec mz: 915.4185 Actual spec prec mz: 915.41847
Fragments matched per AA: 2.46 Proportion of top 20 peaks matched: 0.1
Intensity
6e+06
4e+06
2e+06
0e+00
500
1000
1500
M/Z
y14
y15
B
Scatterplot of predicted elution time
Fitting R2: 0.808
Novel peptide residual Z score: −0.785
Number of peptides: 4551
Pred. hydrophobicity
60
40
20
0
0
20000
40000
60000
Experimental scan number
C
Distributions of residuals from best−fit line
of predicted RT vs Expt. scan number
Line: Z score of novel peptide
Z: −0.785
count
1000
500
0
−10
−5
0
5
10
Z scores of residuals from best linear fit
